# Systemic Sclerosis Dermal Fibroblast Exosomes Trigger Type 1 Interferon Responses in Keratinocytes via a TBK/JAK/STAT Signaling Axis

**DOI:** 10.1002/art.43029

**Published:** 2024-11-12

**Authors:** Jessica Bryon, Christopher W. Wasson, Katja Koeppen, Francesca Chandler, Leon F. Willis, Stefano Di Donato, Elliott Klein, Elton Zeqiraj, Rebecca L. Ross, Francesco Del Galdo

**Affiliations:** ^1^ University of Leeds Leeds United Kingdom; ^2^ Boehringer Ingelheim Ridgefield Connecticut; ^3^ University of Leeds and the National Institute for Health and Care Research Leeds Musculoskeletal Biomedical Research Centre Leeds United Kingdom

## Abstract

**Objective:**

Activation of type I interferon (IFN) response has been shown to correlate with disease activity in systemic sclerosis (SSc). It is currently unknown whether the tissue‐specific type I IFN activation is a consequence of the response observed in blood or rather its source. Exosomes from SSc fibroblasts were recently shown to activate macrophages in vitro. Here, we aimed to determine the source of type I IFN signature in SSc skin biopsies and the potential role of exosomes from SSc dermal fibroblasts in the process.

**Methods:**

Skin biopsies were obtained from the forearms of healthy patients and of those with SSc and processed for dermal fibroblasts and keratinocytes. Exosomes were isolated from healthy and SSc dermal fibroblast supernatants by ultracentrifugation and added to human skin keratinocytes. Keratinocyte transcriptome was analyzed by RNA sequencing (RNA‐seq) analysis. TANK‐binding kinase (TBK) and JAK were inhibited using a small molecule inhibitor (GSK8612) and tofacitinib, respectively.

**Results:**

SSc skin biopsies showed the highest levels of type I IFN response in the epidermal layer. RNA‐seq analysis of keratinocytes transcriptome following exposure to dermal fibroblast exosomes showed strong up‐regulation of IFN signature genes induced by SSc exosomes compared to healthy control. Inhibition of TBK or JAK activity suppressed the up‐regulation of the IFN signature induced by SSc exosomes.

**Conclusion:**

IFN activation of SSc keratinocytes is dependent on their crosstalk with dermal fibroblasts and inducible by extracellular exosomes. Our data indicate that SSc fibroblast exosomes contribute to the type I IFN activation in SSc skin through activation of pattern recognition receptors upstream of TBK.

## INTRODUCTION

Systemic sclerosis (SSc) is a connective tissue disease driven by tissue and vascular fibrosis of the skin and internal organs. Myofibroblasts have been widely characterized as the key cellular elements of tissue fibrosis for more than two decades. In this context, the key role of profibrotic pathways such as transforming growth factor β and Wnt has been dissected in fine detail. More recently, the activation of type I interferon (IFN) pathway has been associated with markers of disease activity and progression in both skin and lung manifestations, but its relationship with fibroblast activation is less clear.^1^, ^2^, ^3^

Immunohistochemistry analysis of SSc skin biopsies has revealed that, although there is a clear sign of type I IFN activation in SSc dermal fibroblasts, the epidermal layer is the major producer of type I IFN response.^2^ The trigger for the response in the epidermis is still unclear. Previous studies have shown cancer‐associated fibroblast (CAF) exosomes can induce IFN‐stimulated gene (ISG) expression in associated epithelial cells.^4^ In particular, CAF exosomes were shown to trigger type I IFN activation through uncapped RNA‐dependent activation of retinoic acid–inducible gene I (RIG‐I) in the epithelial cells, which in turn was dependent on increased activity of RNA polymerase III (Pol III).^5^ These findings are particularly relevant as SSc fibroblasts share a number of similarities with CAFs,^6^, ^7^ and autoantibodies against RNA Pol III are one of the SSc‐specific antinuclear antibodies carrying poor prognostic value for disease progression.^8^ Consistent with these findings, while our studies were in progress, Bhandari et al showed that SSc dermal fibroblast exosomes are able to trigger the activation of macrophages in vitro.^9^ These recent data support the previously observed role of dermal fibroblasts in participating in the pathogenesis of SSc beyond tissue fibrosis^10^ and possibly in a direct activation of the immune events participating to tissue damage.

In this context, we set out to determine whether SSc dermal fibroblasts could participate in the type I IFN response observed in SSc skin, particularly through extracellular vesicles. Here we show for the first time that the type I IFN activation shown by keratinocytes in vivo can be driven by SSc fibroblast exosomes. Furthermore, we show that exosomes induced type I IFN signaling in keratinocytes is dependent upon the exosome RNA induced activation of the TANK‐binding kinase (TBK)1/JAK/STAT signaling cascade.

## MATERIALS AND METHODS

### Patient cell lines

Full thickness skin biopsies were surgically obtained from the forearms of four adult healthy controls and five adult patients with recent onset SSc, defined as a disease duration of less than 18 months from the appearance of clinically detectable skin induration. All patients satisfied the 2013 American College of Rheumatology/EULAR criteria for the classification of SSc^29^. All participants provided written informed consent to participate in the study. Informed consent procedures were approved by NRES‐011NE to author FDG. Fibroblasts were isolated and established as previously described. SSc keratinocytes were grown from dissected skin biopsies and grown in keratinocyte grown media (Promega). Primary cells were immortalized using human telomerase reverse transcriptase (hTERT) to produce healthy control hTERT and SSc hTERT.

### Cell culture

The immortalized keratinocyte cell line HaCaTs were maintained in Dulbecco's modified Eagle medium (DMEM) (Gibco) supplemented with 10% fetal bovine serum (FBS) (Sigma) and penicillin‐streptomycin (Sigma). HaCaTs were stimulated with 1% total volume of healthy and SSc fibroblasts exosomes for 48 hours. TBK1 was inhibited with GSK8612 (10 μM) and JAK1 inhibitor tofacitinib (10 μM) for 48 hrs.

### Transwell assay

Fibroblasts were seeded onto 0.4‐micron pore polyethylene terephthalate transmembranes (Corning). An equal number of HaCaTs were seeded into a separate cell‐culture plate. Upon confluency, the pore was inserted into the well and incubated for 48 hours. The HaCaTs were harvested for RNA and protein analysis. The fibroblasts were stained with DAPI and counted to ensure equal amounts of fibroblasts were used in the experiment.

### Exosome isolation

Fibroblast cell cultures were grown as discussed above with exosome‐depleted FBS. Exosomes were isolated from media collected after 48 to 72 hours by ultracentrifugation (42,000 revolutions per minute for 2 hours). The pelleted exosomes were isolated using the total exosome isolation reagent (Invitrogen) and resuspended in phosphate buffered saline (PBS). RNA was then extracted and purified using the Total Exosome RNA & Protein Isolate Kit (Invitrogen), according to the manufactures protocol.

### Exosomal RNA transfections

Exosomal RNA (50 ng) isolated from healthy or SSc fibroblast exosomes was transfected into HaCaTs using Lipofectamine 2000 (Invitrogen) and incubated for 48 hours. In addition, the exosome RNA was pretreated with RNase A for 2 hours at 37°C, after which the RNase A was inhibited with RNase inhibitor before transfection. Lipofectamine 2000 in the absence of RNA was added to the mock control cells.

### Immunolabeling and visualization of fibroblast‐derived exosomes internalization in human epidermal keratinocytes

Isolated fibroblast exosomes were stained Vybrant DiO cell‐labeling solution (Invitrogen) and added to HaCaT culture media. Exosomes were added at 3, 6, 10, 24, and 48 hours, over a time course. The HaCaTs were imaged on an LMS700 confocal scanning inverted microscope. The images were processed in Image J and Zen 3.1 (blue edition) software.

### CD63 enzyme‐linked immunosorbent assay

Exosome abundance was measured using the ExoELISA‐ULTRA CD63 enzyme‐linked immunosorbent assay (ELISA) (System Biosciences). The ELISA plate is precoated with an anti‐CD63 primary antibody, which recognizes the tetraspanin CD63 on the exosome surface. The secondary antibody contains a horseradish peroxidase enzyme used for signal amplification. The colorimetric substrate 3,3′,5,5′‐tetramethylbenzidine is used for the assay read out. The results are read on a Thermo Scientific Multiskan EX spectrophotometer at a wavelength of 450 nm.

### Dynamic light scattering

Exosomes were diluted in sterile PBS to a concentration of 100 nM. The samples were injected (150 μL, per sample) into a Wyatt miniDawn Treos system (equipped with an additional dynamic light scattering [DLS] detector). The raw data were analyzed using the ASTRA 6.0.3 software supplied by the instrument, with the regularization algorithm used to generate the size‐distribution histograms. The histograms of signal intensity versus hydrodynamic radius were then plotted in OriginLab.

### Western blotting

Total proteins were extracted from fibroblasts in radioimmunoprecipitation assay buffer and resolved by sodium dodecyl sulfate–polyacrylamide gel electrophoresis (10%–15% Tris‐Glycine). Proteins were transferred onto Hybond nitrocellulose membranes (Amersham) and probed with antibodies specific for pSTAT1, total STAT1 (Cell Signaling Technology), CD63 (Abcam), TSG101(Abcam), histone 3 (Cell Signaling Technology), pIRF3 (Abcam), IRF3 (Cell Signaling Technology), and β‐Actin (Sigma). Immunoblots were visualized with species‐specific horseradish peroxidase (HRP)‐conjugated secondary antibodies (Sigma) and enhanced chemiluminescence (Thermo Scientific/Pierce) on a Bio‐Rad ChemiDoc imaging system.

### RNA sequencing

Quality control of fastq files was performed with FastQC version 0.11.9. Reads were aligned to the human reference genome version GRCh38.84 using STAR aligner version 2.5.2b.^13^ MultiQC version 1.11^14^ was used to perform quality control on the alignment and generate an overall quality control report. Downstream analyses and visualizations were performed using R version 4.1.2 (https://www.R-project.org/). BAM alignment files generated by STAR were transformed into count tables using Rsubread version 2.8.1^15^.The R package edgeR version 3.36.0^16^ was used for differential gene expression analysis. Gene set enrichment analysis for gene ontology biologic processes was performed using WebGestalt^27^, ^28^.

### Quantitative reverse transcription–polymerase chain reaction

RNA was extracted from cells using commercial RNA extraction kits (Zymo Research). RNA (1 μg) was reverse transcribed using complementary DNA synthesis kits (ThermoFisher Scientific). Quantitative reverse transcription–polymerase chain reactions (qRT‐PCRs) were performed using SyBr Green PCR kits on a Thermocycler with primers specific for MX1 (forward: CGACACGAGTTCCACAAATG reverse: AAGCCTGGCAGCTCTCTACC), CXCL10 (forward; GGTGAGAAGAGATGTCTGAATCC reverse; GTCCATCCTTGGAAGCACTGCA), CXCL11 (forward; TCCCCCATGTTCAAAAGAGGAC reverse; ATATCTGCCACTTTCACTGCTTTTAC), OAS1 (forward; CGGACCCTACAGGAAACTTG reverse; GAAGCAGGAGGTCTCACCAG), IFIT1 (forward; GACTGGCAGAAGCCCAGACT reverse; GCGGAAGGGATTTGAAAGCT) and GAPDH (forward; ACCCACTCCTCCACCTTTGA reverse; CTGTTGCTGTAGCCAAATTCGT). Data were analyzed using the ΔΔ Ct method. GAPDH served as a housekeeping gene. Type I IFN superarray was performed using manufacturer protocol (Qiagen).

### Transmission electron microscopy

Healthy and SSc exosomes were loaded onto carbon‐coated copper grids (Formvar/Carbon, 300 mesh Cu, Agar Scientific). Grids were glow discharged for 30 seconds at 0.39 mBar pressure and 10 mA (PELCO easiGlow, Ted Pella). Grids were incubated for 1 minute with a 7‐μL sample, washed three times with ddH_2_O, and stained with 2% (weight/volume) uranyl acetate. Data were collected using a Tecnai F20 transmission electron microscope (FEI) at 200 KeV, fitted with a Ceta CMOS CCD camera (FEI). Micrographs were collected at 40,000× magnification with a pixel size of 3.51 Å. Image J was used to add a scale bar and quantify the size of the vesicles.

### Immunohistochemistry

Immunohistochemistry was performed as previously described.^2^ Sections were stained with a pSTAT1 (Cell Signaling Technology 1/200), MX1 (Abcam 1/100), and CXCL10 antibody (Abcam 1/200), visualized using an HRP‐conjugated mouse secondary, and counterstained with hematoxylin.

### Statistical analysis

Data are presented as the mean ± SE. Statistical analysis was performed using a two‐tailed, paired Student's *t*‐test for singular analysis and analysis of variance tests for multicomparison analysis.

### Ethics approval and consent to participate

The study was approved by National Research Ethics service (NRES) committee North East‐Newcastle & North Tyneside: REC Ref: 15/NE/0211 to FDG. All participants provided written informed consent to participate in this study. Informed consent procedure was approved by NRES‐011NE to FDG by the University of Leeds.

## RESULTS

### Loss of SSc keratinocyte expression of type 1 IFN signature in vitro and induction by SSc dermal fibroblast coculture

**Figure 1:**
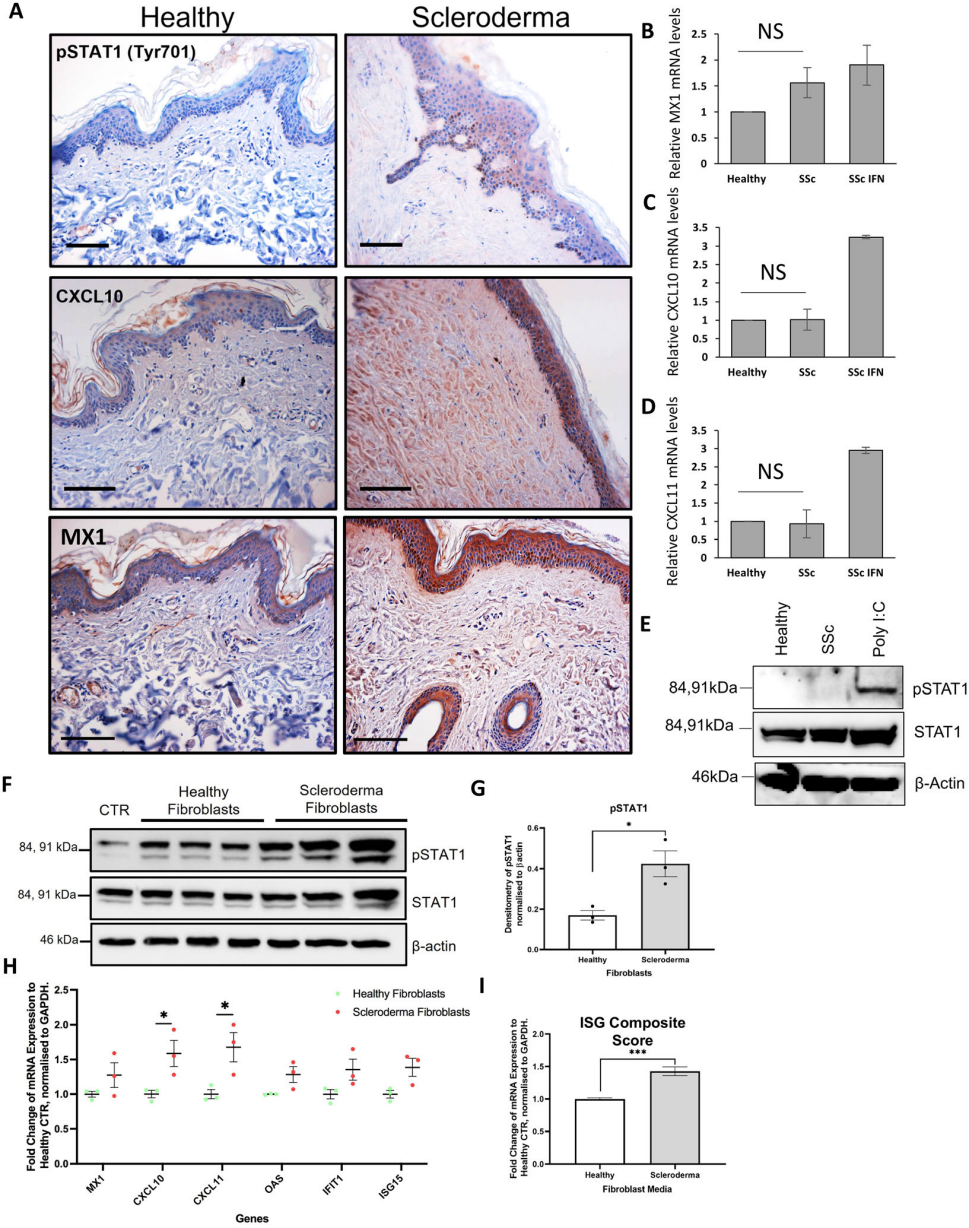
Scleroderma dermal fibroblasts induce type I IFN signaling in keratinocytes. (A) Healthy (N = 5) and SSc (N = 5) skin sections obtained from patient forearms were stained with antibodies specific for phosphorylated STAT1, CXCL10, and MX1. Scale bars represent 50 μm. (B–D) SSc skin keratinocytes were isolated from skin biopsies and passaged three times; RNA was then isolated. SSc keratinocytes were stimulated with IFN‐α (2 ng/mL) for 2.5 hours. (B) MX1, (C) CXCL10, and (D) CXCL11 transcript levels were assessed by qRT‐qPCR. Graphs represent fold change relative to healthy control. (E) Protein was isolated from SSc keratinocytes (n = 3) and healthy (n = 3) control keratinocytes. Lysates from healthy keratinocytes stimulated with POLY I:C (24 hours 10 μg/mL) was used as a positive control. pSTAT1 and total STAT1 protein levels were assessed by Western blot. β‐actin was used as a loading control. (F and G) Healthy (n = 3) and scleroderma (n = 3) fibroblasts were cocultured in cell‐culture inserts with human epidermal keratinocytes (HaCaTs) for 48 hours in serum‐free DMEM. (F) pSTAT1 (Tyr701) and STAT1 protein levels were assessed by Western blot. β‐actin was used as a loading control. (G) Protein expression of pSTAT1 was quantified by densitometry and normalized to β‐actin levels. (H) Fold change of mRNA transcript expression of ISGs (MX1, CXCL10, CXCL11, OAS, IFIT1, and ISG15) in HaCaTs cocultured with healthy fibroblasts, compared to SSc. (I) Fold change of mRNA transcript expression of an ISG composite score of the six genes described in H genes, normalized to GAPDH and ribosomal protein as a housekeeping gene, relative to mean healthy exosome‐treated HaCaTs. *P < 0.05; ***P < 0.001. CTR, Control; DMEM, Dulbecco's modified Eagle medium; IFN, interferon; ISG, IFN‐stimulated gene; mRNA, messenger RNA; NS, not significant; RT‐qPCR, quantitative reverse transcription–polymerase chain reaction; SSc, systemic sclerosis.

Previous studies, including our own, have shown that keratinocytes in SSc skin are a major source of ISG expression.^2^ Consistent with these findings, we observed high levels of pSTAT1, CXCL10, and MX1 in the epidermis of SSc skin compared to healthy control biopsies (Figure 1A). We then set out to determine whether SSc keratinocytes could maintain the ISG response when isolated from the skin, similarly to what has been repeatedly observed for the profibrotic activation of dermal fibroblasts.^17^ SSc keratinocytes isolated from the same biopsies shown in Figure 1A did not display elevated MX1, CXCL10, or CXCL11 transcript levels or pSTAT1 protein levels (Figure 1B–E), compared to healthy control keratinocytes, when cultured in vitro. Interestingly, these isolated SSc keratinocytes were still able to induce ISG expression upon stimulation with IFN‐α (Figure 1C and D), suggesting the type I IFN signaling pathway is still functional in the SSc keratinocytes in culture.

Next, we assessed whether SSc fibroblasts could trigger type I IFN response in keratinocytes. To focus our study on the role of the fibroblasts we cocultured the skin keratinocyte cell line HaCaTs with three different healthy and SSc fibroblasts in a transwell assay (Figure 1F). We observed that both healthy and SSc dermal fibroblasts triggered STAT1 phosphorylation in HaCaTs compared to the control, with a significantly greater induction in the HaCaTs cultured with SSc dermal fibroblasts (Figure 1G). Accordingly, SSc dermal fibroblasts induced greater levels of messenger RNA (mRNA) expression of ISGs (MX1, CXCL10, CXCL11, OAS1, IFIT1, and ISG15) compared to healthy control (Figure 1H; Supplementary Figure 1). Combining the ISG analyzed in a composite score as previously described,^2^ we observed an overall 1.5‐fold increase of ISG induction in the HaCaTs cocultured with SSc fibroblasts compared to healthy control (Figure 1I).

### Characteristics of healthy and SSc fibroblast exosomes

**Figure 2:**
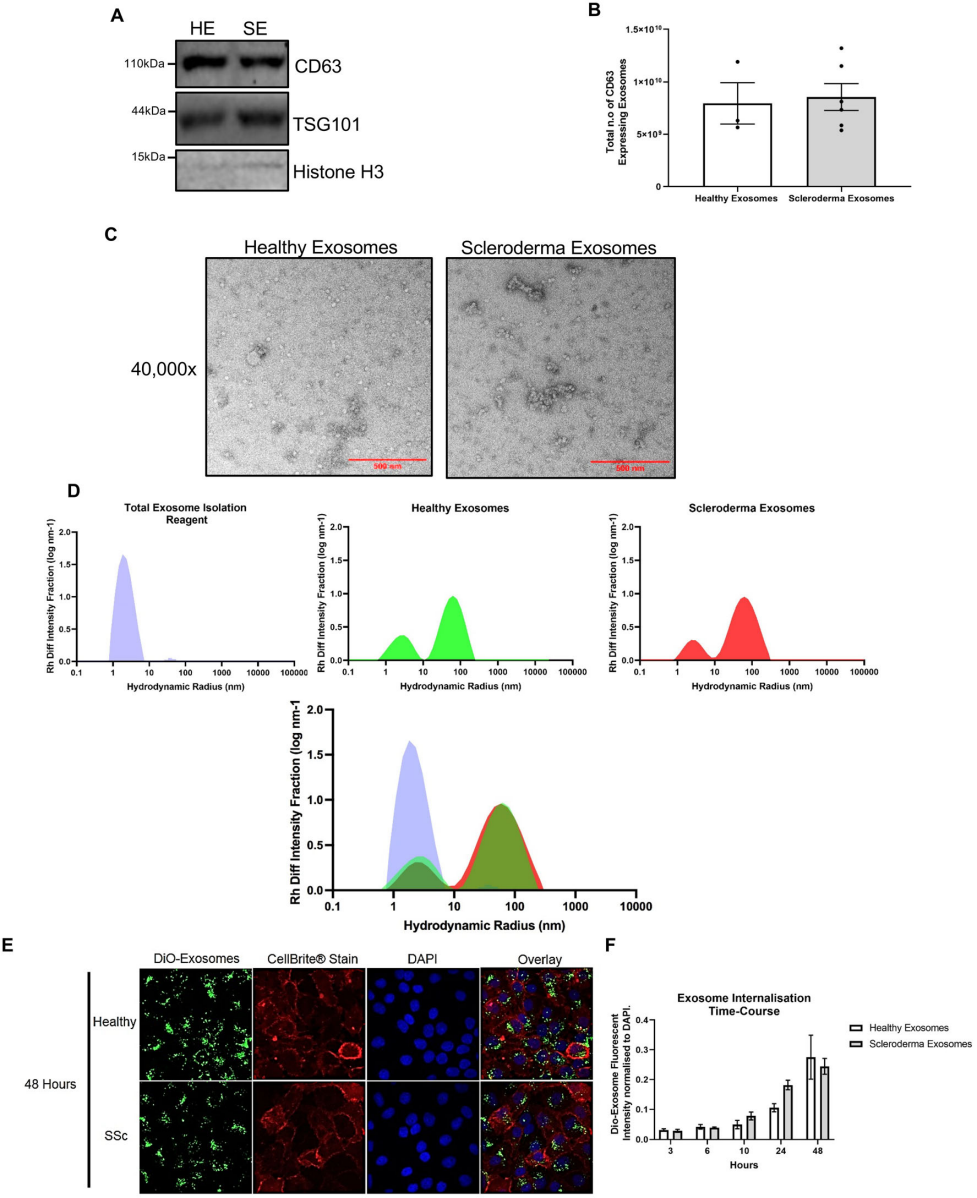
Characterization of exosomes from healthy SSc dermal fibroblasts. Exosomes were isolated from healthy (n = 3) and SSc (n = 6) fibroblast media. (A) Isolated exosomes were probed for TSG101, CD63, and histone H3 by Western blot. (B) CD63 ELISA quantification of exosomes from healthy (n = 3) and SSc (n = 5) patient fibroblasts. (C) Representative transmission electron microscopy images of healthy and scleroderma exosomes at 40,000× magnification. (D) Representative size‐distribution histograms of diluted total exosome isolate buffer, healthy (n = 3) and SSc (n = 3) exosomes, from regularization analysis of dynamic light scattering data. (E) Healthy and SSc fibroblast exosomes were stained with DiO‐lipid dye and added to HaCaTs at 3, 6, 10, 24, and 48 hours. (F) Bar charts illustrates the green, fluorescent intensity per image, normalized to include a DAPI intensity, per three images over a time course (3, 6, 10, 24, and 48 hours). ELISA, enzyme‐linked immunosorbent assay; HE, healthy exome; Rh diff Hydrodynamic Radius; SSc, systemic sclerosis; SE, SSc exome.

To determine whether fibroblast exosomes could contribute to the induction of type I IFN in keratinocytes, we isolated exosomes from healthy and SSc fibroblast supernatants. Firstly, we observed that healthy and SSc fibroblasts secreted similar amounts of exosomes as shown by similar levels of exosome marker expression (TSG101 and CD63) in the isolated exosomes (Figure 2A). This was confirmed via CD63 ELISA whereby we quantified that both healthy and SSc fibroblasts secreted exosomes in the order of 1 × 10^10^ (from 20 million fibroblasts) (Figure 2B). Furthermore, electron microscopy analysis and DLS indicated that the exosomes secreted from healthy and SSc fibroblasts were of similar size and distribution (Figure 2C and D). Finally, we set out to determine the cell entry kinetics of both sets of exosomes as assessed by membrane‐specific dye staining followed by immunofluorescence analysis. As shown in Figure 2E, we observed no apparent difference in timing and intracellular localization of fibroblast exosomes from SSc or healthy control dermal fibroblasts, with a time‐dependent increase in exosome uptake continuing to rise up to the 48 hours in both sets (Figure 2F).

### Proinflammatory effect of SSc fibroblast exosomes on keratinocytes compared with healthy control fibroblast exosomes

**Figure 3:**
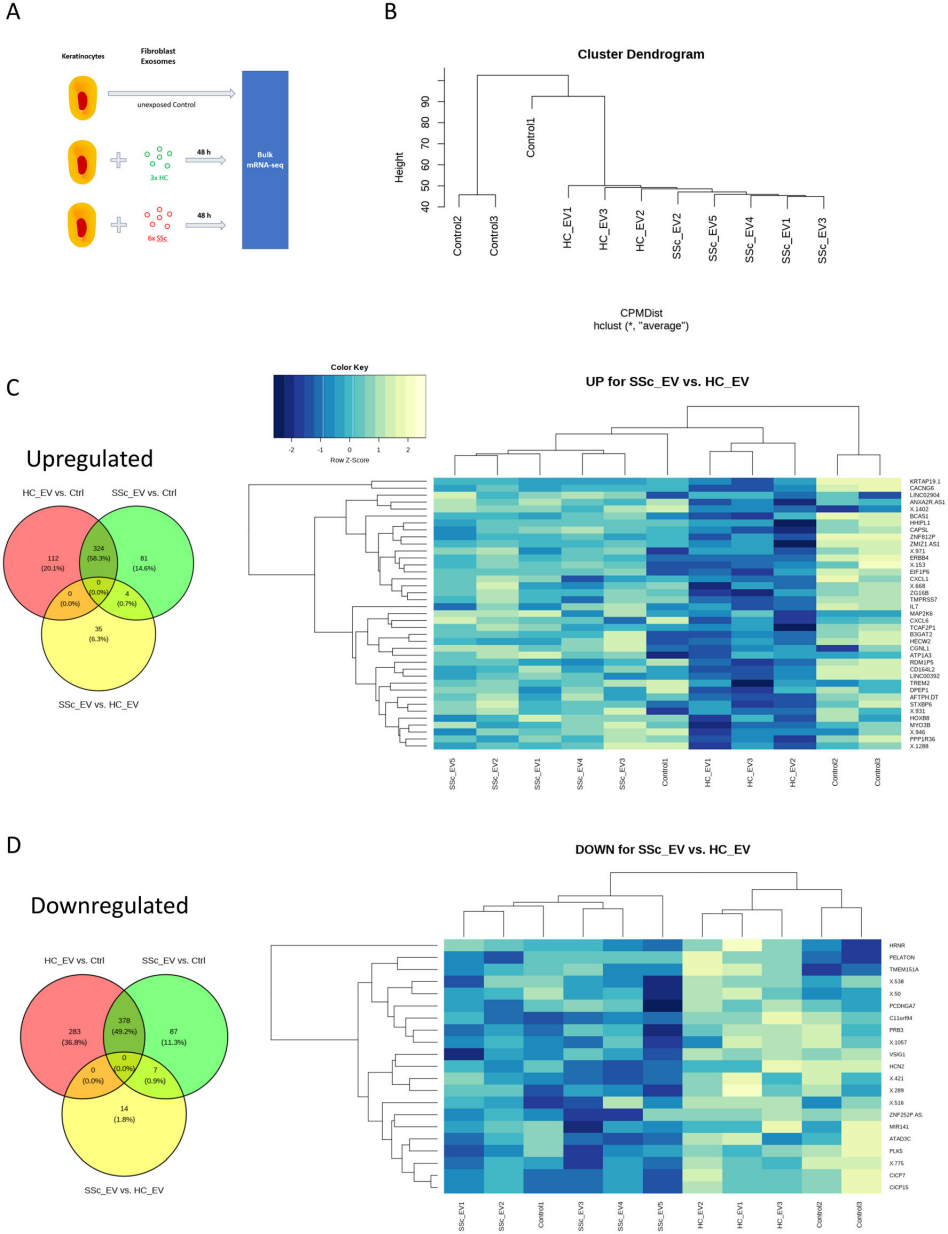
Global gene analysis of keratinocytes stimulated with healthy and SSc dermal fibroblast exosomes. (A) Schematic representation of experimental approach for the RNA‐seq analysis. HaCaT keratinocytes were stimulated with healthy (n = 3) and SSc (n = 5) fibroblast exosomes for 48 hours. RNA was isolated from the stimulated HaCaTs, and RNA‐seq analysis was performed. (B) Cluster dendrogram clustering of the responses of healthy and SSc in HaCaTs compared to control. Venn diagram and heatmaps of differential expression genes with a fold change greater than 1.5‐fold scaled by row for (C) genes up‐regulated and (D) genes down‐regulated in HaCaTs stimulated with SSc fibroblast exosomes compared to healthy fibroblast exosomes. The heat map illustrates significantly (*P* < 0.05) differentially expressed genes and the color scale represents the row Z‐score, with dark blue representing low gene expression levels and light yellow indicating high expression relative to other samples in the same row. ctrl, control; EV, Extracellular Vesicles; HC, healthy control; mRNA‐seq, messenger RNA sequencing; RNA‐seq, RNA sequencing; SSc, systemic sclerosis.

To determine the global effects of the SSc fibroblast exosomes on skin keratinocytes, we performed RNA sequencing (RNA‐seq) of HaCaTs following a 48‐hour culture in exosome conditioned media (1% total volume) (Figure 3A). Hierarchical clustering of differentially expressed genes (DEGs) showed a clear separation of the transcriptomic response induced by exosomes compared to mock and a separation between SSc and healthy exosomes (Figure 3B). The RNA‐seq analysis revealed there were 1,097 DEGs (*P* < 0.05 and abs (log2 FC) > 1) between HaCaTs stimulated with healthy fibroblast exosomes compared to control HaCaTs, 881 DEGs in HaCaTs stimulated with SSc fibroblast exosomes compared to control HaCaTs, and 60 DEGs in HaCaTs stimulated with SSc fibroblast exosomes compared to HaCaTs stimulated with healthy fibroblasts exosomes. Within the 60 SSc‐specific DEGs found, 39 were up‐regulated (Figure 3C) and 21 were down‐regulated (Figure 3D) in keratinocytes stimulated with SSc fibroblast exosomes compared to healthy control. In addition, Supplementary Table 1 contains a list of the top 60 DEGs (fold change) found in HaCaTs stimulated with SSc fibroblast exosomes compared to healthy fibroblast exosomes.

**Figure 4:**
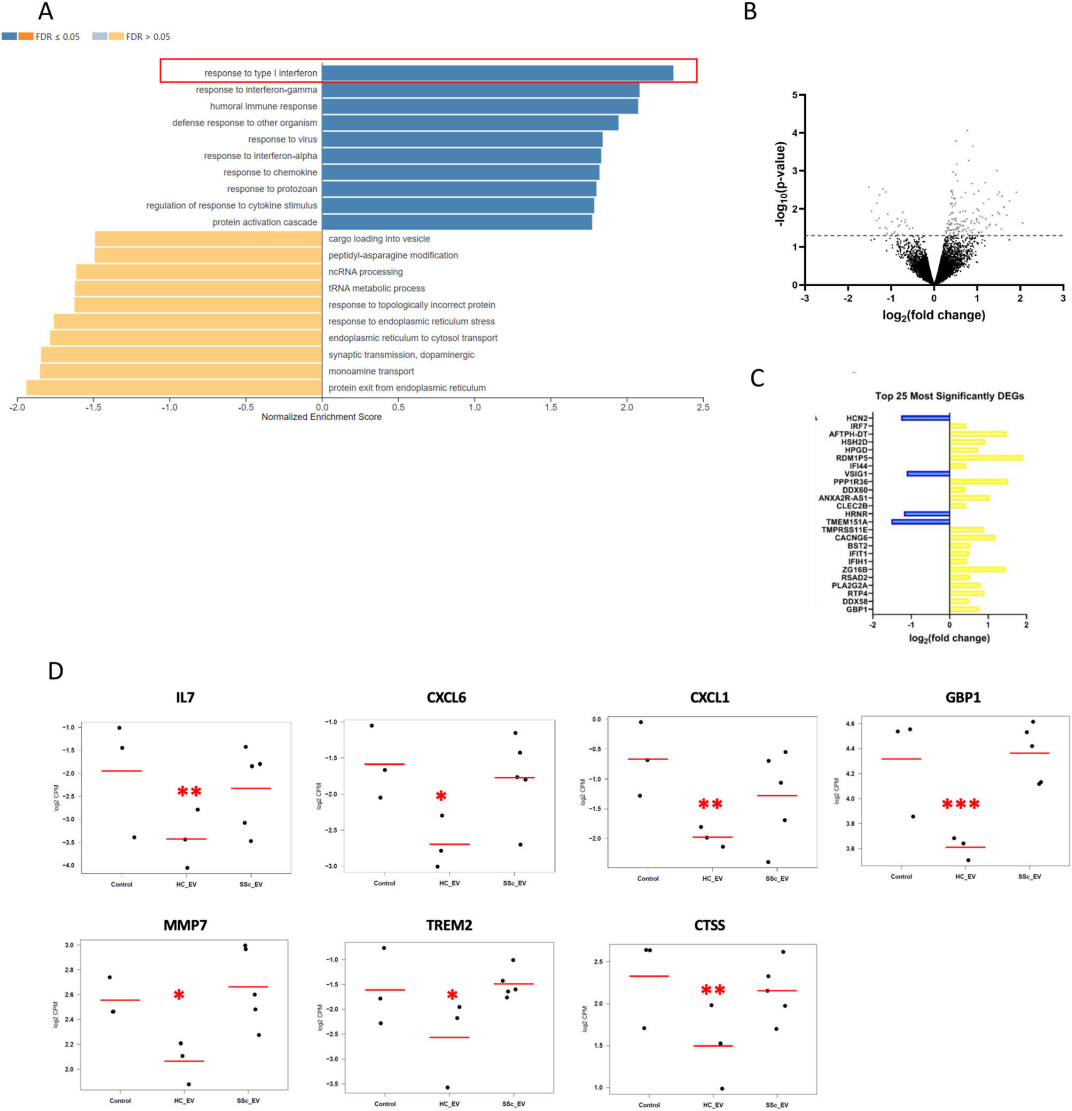
SSc fibroblast exosomes impart a proinflammatory effect on keratinocytes compared to healthy control fibroblast exosomes. (A) Gene ontology enrichment analysis of biologic processes associated with DEGs, in HaCaTs treated with SSc exosomes compared to healthy control exosomes. Dark blue or orange = FDR < 0.05; light blue or orange FDR > 0.05. (B) Volcano plot of log2 fold change against‐log10 *P* value for each DEG in HaCaTs stimulated with SSc fibroblast‐derived exosomes, when compared against healthy exosomes. Red = significant DEGs. Blue = significant DEGs associated with interferon signaling. (C) A list of the 25 most significant DEG from the analysis. (D) Gene expression levels of selected ISGs (IL‐7, CXCL6, CXCL1, GBP1, MMP7, TREM2, and CTSS). Comparisons made between control HaCaTs and HaCaTs stimulated with healthy and SSc dermal fibroblast exosomes. *P < 0.05; **P < 0.01; ***P < 0.001. DEG, differentially expressed gene; FDR, false discovery rate; IL, interleukin; ISG, interferon‐stimulated gene; ncRNA, noncoding RNA; SSc, systemic sclerosis; tRNA, transfer RNA.

Gene ontology enrichment analysis using the fold changes for all detected genes revealed that several pathways involved in immune signaling (responses to type 1 IFN, responses to IFN‐γ, humoral immune responses, and response to chemokines) were up‐regulated in HaCaTs stimulated with SSc fibroblast exosomes compared to healthy fibroblast exosomes (Figure 4A). Analysis of individual genes showed a number of ISGs were differentially up‐regulated in HaCaTs stimulated with SSc fibroblast exosomes, as shown in the volcano plot (Figure 4B). A list of the 25 most significant DEGs is shown in Figure 4C. The list includes a number of ISGs. Further analysis of type I IFN signaling in HaCaTs stimulated with healthy and SSc dermal fibroblast exosomes compared to the mock control revealed that the healthy dermal fibroblast exosomes impart an anti‐inflammatory response on the keratinocytes as shown by reduced expression of a number of ISGs. Expression levels of interleukin (IL)‐7, CXCL6, CXCL1, GBP1, MMP7, TREM2, and CTSS were significantly reduced in HaCaTs stimulated with healthy fibroblast exosomes compared to unexposed HaCaTs (Figure 4D). Expression levels of these ISGs were also significantly reduced in HaCaTs stimulated with healthy control fibroblasts compared to HaCaTs stimulated with SSc fibroblasts and not significantly different between HaCaTs stimulated with SSc dermal fibroblast exosomes and unexposed control HaCaTs (Supplemental Figure 2). Collectively, these data suggest that an important function of exosomes in healthy skin is to dampen proinflammatory IFN signaling in keratinocytes, thus contributing to homeostasis, and that the lack of this anti‐inflammatory effect of exosomes secreted by SSc fibroblasts on keratinocytes may lead to loss of homeostasis and contribute to a more proinflammatory state in patients with SSc.

**Figure 5:**
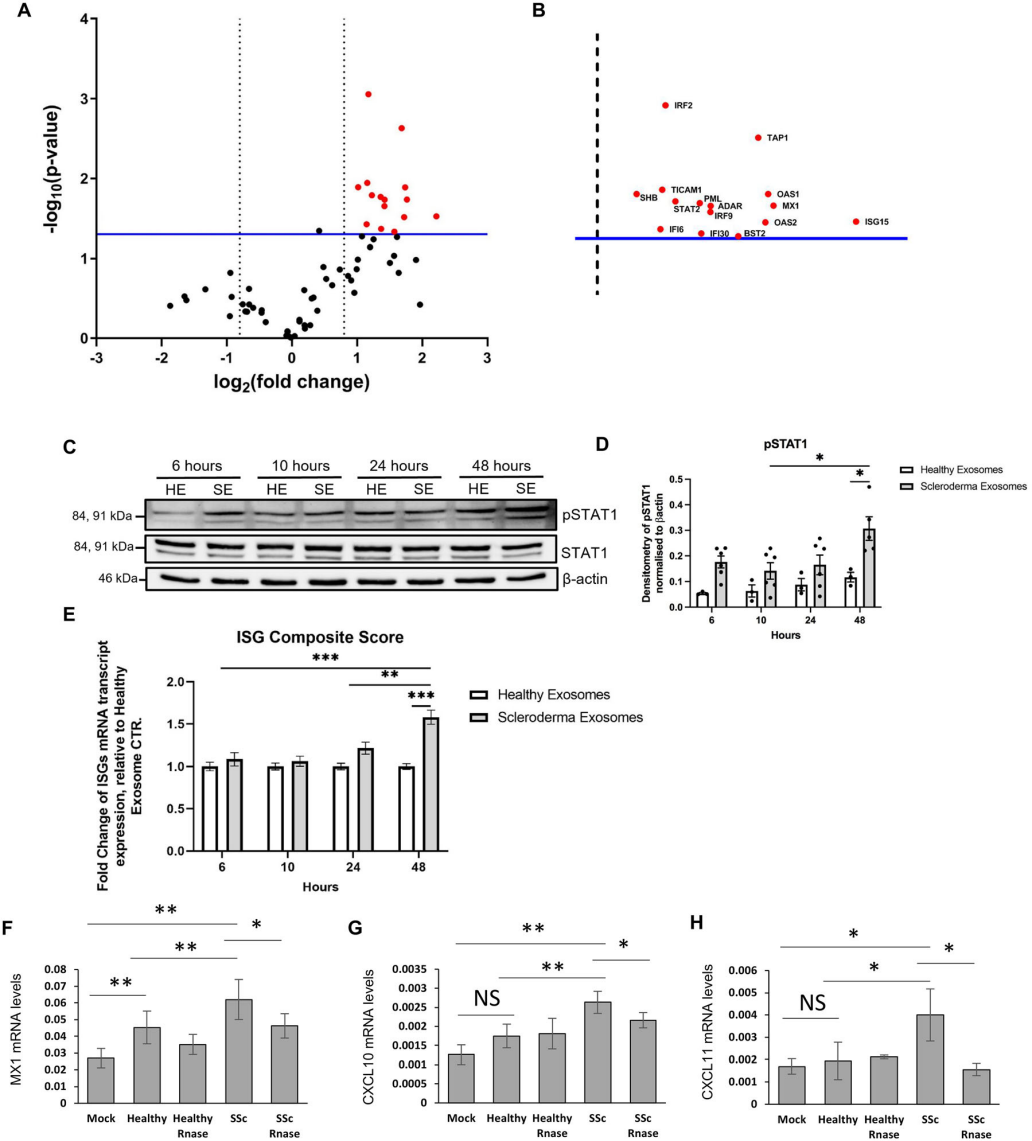
Scleroderma dermal fibroblast exosomes induce type 1 IFN signaling in keratinocytes through their RNA content. HaCaT keratinocytes were stimulated with healthy and SSc fibroblast exosomes for 48 hours. RNA was extracted from the stimulated HaCaTs. A type 1 IFN superarray was performed. (A) Volcano plot which shows the fold change of gene expression of IFN type 1–stimulated genes in HaCaTs stimulated with SSc exosomes relative to HaCaTs stimulated with healthy exosomes. (B) An enlarged version of the volcano plot in illustrating the identifiers of the genes significantly up‐regulated in SSc versus healthy exosome‐treated HaCaTs. HaCaT keratinocytes were stimulated with healthy and SSc fibroblast exosomes for 6 to 48 hours. Protein and RNA were isolated from stimulated HaCaTs. (C) pSTAT1 (Tyr701) and STAT1 protein levels were assessed by Western blot. β‐actin was used as a loading control. (D) Bar chart illustrates quantification of protein expression by densitometry and normalized to β‐actin levels. Data represent means ± SE. Data were checked for normality and statistically analyzed using a two‐way multiple comparison ANOVA, normalized by Tukey. (E) Bar chart illustrates the fold change of mRNA transcript expression of an ISG composite score of multiple IFN‐related genes normalized to GAPDH, relative to healthy exosome stimulations, over a time course. ISG composite score included 6 ISGs: MX1, CXCL10, CXCL11, OAS, IFIT1, and ISG1. Data represent means ± SE. Data were checked for normality and statistically analyzed using a two‐way multiple comparison ANOVA, normalized by Tukey. RNA was extracted from healthy and SSc fibroblast exosomes and transfected into HaCaT cells for 48 hours. In addition, the RNA was pretreated with RNase before transfection. (F) MX1, (G) CXCL10, and (H) CXCL11 transcript levels were assessed by RT‐qPCR. *P < 0.05; **P < 0.01; ***P < 0.001. ANOVA, analysis of variance; CTR, Control; IFN, interferon; ISG, IFN‐stimulated gene; mRNA, messenger RNA; NS, not significant; RT‐qPCR, quantitative reverse transcription–polymerase chain reaction; SSc, systemic sclerosis.

Previous studies have shown SSc fibroblast^9^ and CAF exosomes^4^, ^5^ actively stimulate macrophages and epithelial cells compared to healthy control fibroblast exosomes. Here we show healthy dermal fibroblast exosomes negatively regulate type I IFN signaling and this ability is lost in SSc fibroblasts, causing an increase type I IFN signature in HaCaTs relative to healthy control exosomes. To validate this finding, we performed a qRT‐PCR–based superarray to assess 79 type I IFN–dependent ISGs on RNA isolated from HaCaTs treated with healthy and SSc dermal fibroblast exosomes. Volcano plot analysis on three independent experiments showed 16 ISGs up‐regulated by SSc exosomes compared to healthy exosomes (Figure 5A and B; Supplementary Figure 3). Altogether, the qRT‐PCR–based IFN array validated the RNA‐seq data, indicating a strong type I IFN response in HaCaTs stimulated with SSc fibroblast exosomes compared to healthy control exosomes. At the protein level, whole‐cell protein lysates from the HaCaTs in the same experimental conditions showed a time‐dependent increase in pSTAT1 levels (Figure 5C and D), consistent with the RNA findings (Figure 5A–B) and the kinetics of exosome internalization (Figure 2E). Accordingly, the ISG composite score showed a similar increment over time, becoming significantly induced by SSc exosomes at 24 and 48 hours (Figure 5E).

### Type 1 IFN signaling in keratinocytes from SSc fibroblast exosomes through a TBK1/JAK‐dependent response to their RNA cargo

Previous studies have shown that specific uncapped RNA transcripts within CAF exosomes can trigger type I IFN signaling in epithelial cells^5^.^9^ Therefore, we wanted to determine if the RNA contained within the SSc fibroblast exosomes contributed to the type I IFN stimulation observed in keratinocytes. RNA was extracted from healthy and SSc dermal fibroblast exosomes and transfected into HaCaT cells (50 ng RNA). Healthy fibroblast exosome RNA had minimal effects on MX1, CXCL10, and CXCL11 expression in HaCaTs compared to control cells. SSc fibroblast exosome RNA induced a 2.5‐fold increase in MX1 (*P* = 0.002), 2‐fold increase in CXCL10 (*P* = 0.0015), and 3‐fold increase in CXCL11 (*P* = 0.016) expression in HaCaTs (Figure 5F–H). Pretreatment of exosome extracts with RNase significantly suppressed the up‐regulation of MX1, CXCL10 and CXCL11, although with variable efficiency (Figure 5F–H). This suggests that the RNA within the exosomes directly contributed to type I IFN response of HaCaT, in vitro.

**Figure 6:**
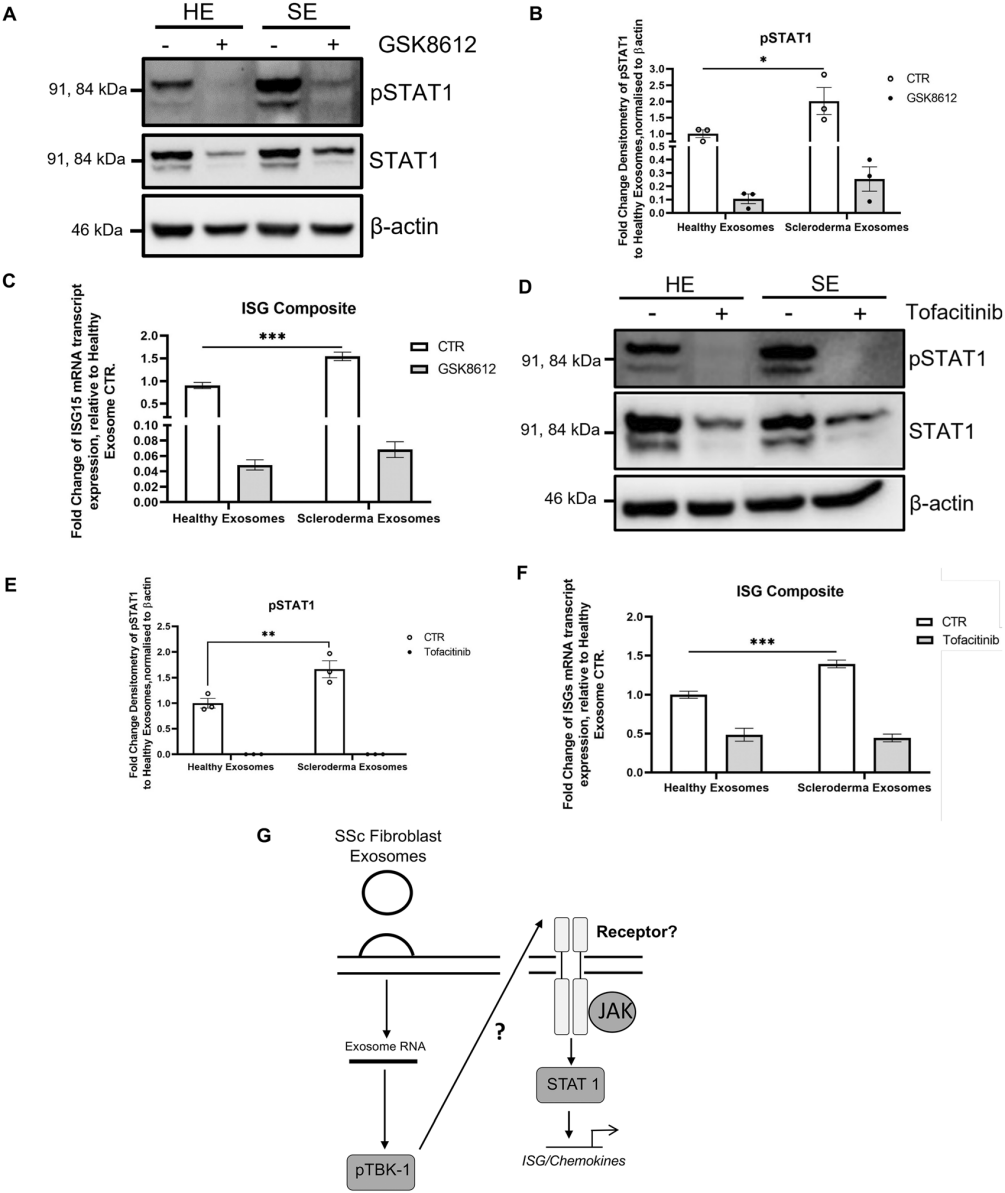
Inhibition of TANK‐binding kinase and JAK‐STAT blocks SSc fibroblast exosome–mediated type I IFN signaling in HaCaTs. (A) HaCaTs were stimulated with healthy and SSc fibroblast exosomes for 48 hours. In addition, the HaCaTs were treated with 10 μM GSK8612, one hour before stimulation. pSTAT1 and STAT1 protein levels were assessed by Western blot. β‐actin was used as a loading control. (B) The bar chart illustrates densitometry analysis of pSTAT1. Data represent means ± SE from three biologic repeats. (C) Fold change of mRNA transcript expression of multiple IFN‐related genes, normalized by housing keeping gene GAPDH, and relative to untreated healthy exosome‐treated HaCaTs. Data represent means ± SE from three biologic repeats (healthy) and four biologic repeats (SSc). ISG composite score included 6 ISGs: MX1, CXCL10, CXCL11, OAS, IFIT1, and ISG15. Statistical analysis was performed using a multiple comparison two‐way ANOVA, normalized to Tukey. (D) HaCaTs were stimulated with healthy and SSc exosomes for 48 hours. In addition, the HaCaTs were treated with 20 μM tofacitinib one hour before stimulation. pSTAT1 and STAT1 protein levels were assessed by Western blot. β‐actin was used as a loading control. (E) The bar chart illustrates densitometry analysis of pSTAT1. Data represent means ± SE from three biologic repeats. (F) Fold change of mRNA transcript expression of multiple IFN‐related genes, normalized by housing keeping gene GAPDH, and relative to untreated healthy exosome‐treated HaCaTs. Data represent means ± SE. ISG composite score included six ISGs: MX1, CXCL10, CXCL11, OAS, IFIT1, and ISG15. Statistical analysis was performed using a multiple comparison two‐way ANOVA, normalized to Tukey. (G) Schematic representation of the mechanism behind SSc fibroblast exosome induction of type I IFN signaling. *P < 0.05; **P < 0.01; ***P < 0.001. ANOVA, analysis of variance; CTR, Control; HE, healthy exome; IFN, interferon; ISG, IFN‐stimulated gene; mRNA, messenger RNA; SE, SSc exome; SSc, systemic sclerosis.

There are a number of ways RNA cargo could induce a type I IFN response, such as a microRNA (miRNA)‐induced up‐regulation of type I IFN or a direct stimulation of RNA sensors, as has been described for CAF‐derived exosomes.^4^, ^5^ Most RNA pattern recognition receptors (TLR‐7, RIG‐I, and MDA5) signal through TBK1. Therefore, we used the specific TBK1 inhibitor GSK8612 to interrogate the pathway in response to exosome stimulation. The observed increased STAT1 activation by SSc fibroblast exosomes was completely abolished with the TBK1 inhibitor (Figure 6A and B). Accordingly, TBK1 inhibition also suppressed the induction of ISG expression in the HaCaTs (Figure 6C). Taken together, this suggests the SSc fibroblast exosomes induce type I IFN signaling in HaCaTs through TBK1. TBK1 is known to activate STAT1 through IRF3/7 in response to viral infections.^18^ Interestingly, the SSc fibroblast exosomes did not activate IRF3 in keratinocytes (Supplementary Figure 4). This suggests that TBK1 activates STAT1 through an alternative pathway. This was further highlighted in the type 1 IFN array and RNA‐seq data in which IFN‐α/β expression was not induced by the SSc fibroblast exosomes.

To determine whether the TBK1 induced activation of STAT was JAK dependent, we used the JAK1 inhibitor tofacitinib in the same experimental conditions of Figure 6A and B. Similar to TBK inhibition, tofacitinib completely blocked the SSc fibroblast exosome–mediated STAT1 activation in HaCaTs (Figure 6D and E) as well as ISG expression (Figure 6F), suggesting that the observed type I IFN response is mediated by activation of JAK. Neither GSK8612 nor tofacitinib affected total STAT1 levels in unstimulated HaCaTs (Supplementary Figure 5). In addition, we observed undetectable levels of pSTAT1 in the unstimulated HaCaTs. Taken together with the data in Figure 6, it suggests that healthy fibroblasts exosomes can induce pSTAT1 levels compared to unstimulated HaCaTs but that SSc fibroblast exosomes further enhance this induction of pSTAT1 in the HaCaTs.

### Analysis of mRNA content between healthy and SSc fibroblast exosomes

In the data above we have shown that SSc dermal fibroblast exosomes can induce a type 1 IFN response in keratinocytes through the TBK1/JAK signaling axis. Further analysis showed that the RNA within the exosomes can induce the type 1 IFN response (Figure 5F–H). We next wanted to identify potential RNA transcripts within the SSc fibroblast exosomes that induce the response. We extracted RNA from healthy and SSc fibroblast exosomes and performed RNA‐seq analysis to assess differential mRNA abundance in SSc compared to healthy control exosomes. Hierarchical clustering of 253 genes that were significantly up‐regulated in the SSc fibroblast exosomes compared to the healthy control showed a clear separation between groups (Supplementary Figure 6A). Individual plots for the eight most highly up‐regulated abundant transcripts (>5 counts per minute) are shown in Supplementary Figure 6B. Pathway analysis of the DEGs did not identify any IFN‐related pathways, but AFF3 and H19, which are two of the most significant DEGs, have tentatively been implicated in immune responses in other autoimmune conditions including rheumatoid arthritis.^19^, ^20^

## DISCUSSION

In this study we have shown the type I IFN response observed in SSc skin is mainly driven by keratinocytes. Importantly, we also show that keratinocytes are “responding” to a primary trigger in their microenvironment as they lose any sign of type I IFN activation once cultured in vitro. Parallel to this evidence, coculture experiments clearly indicate that SSc dermal fibroblasts are able to induce type I IFN activation in keratinocytes, which can be induced by isolated exosomes. These data suggest that dermal fibroblasts may participate to the pathogenesis of SSc not only for their profibrotic activation but also in triggering and/or sustaining the type I IFN response observed in the tissue. Furthermore, these data also suggest that the increase in type I IFN–inducible chemokines observed in peripheral blood may be a consequence of a tissue driven type I IFN response rather than its source. Similar data have been suggested in the pathogenesis of systemic lupus erythematosus.^21^ This interpretation would also be consistent with the observation that serum proteome correlated with skin transcriptome better than peripheral blood transcriptome in SSc.^22^ The transfection of exosome cargo RNA in human keratinocytes suggested that the RNA contained within SSc exosomes is contributing to, if not driving, the type I IFN response. Finally, we interrogated the signaling pathway behind this response with small molecule inhibitors. Although a fine dissection of the pathway(s) fell outside the scope of this study, we were able to demonstrate that SSc fibroblast exosomes induce ISG expression through a TBK1/JAK/STAT1 signaling cascade (Figure 6G).

We believe that these data contribute to identifying the source of type I IFN activation in SSc skin, but we also acknowledge that there are still a number of unanswered questions regarding this phenomenon. Specifically, we still do not have a clear understanding of the factors downstream of TBK1. One standout candidate is IRF3/7. TBK1 has been shown to phosphorylate and activate IRF3/7 in response to viral and bacterial infections, which in turn drives a type I IFN response.^18^ Interestingly, we have shown that SSc fibroblasts exosomes do not activate IRF3 (Supplementary Figure 4). This suggests TBK1 induces ISG expression through alternative downstream targets. One possible target is NF‐κB, which is known to be a downstream target of TBK1.^23^ This hypothesis will be explored in future studies.

The specific exosome cargo that triggers the type I IFN response in the keratinocytes is not fully understood. We have shown that exosome RNA can induce ISG expression in the keratinocytes, but this does not rule out the possibility that proteins or other second messengers in the cargo could also play a role. The exosomes may contain cytokines, which upon exosome uptake could trigger the signaling cascade we have described in the results. Analysis of mRNA content between healthy and SSc fibroblast exosomes revealed few target mRNA differentially expressed in the SSc fibroblast exosomes that may contribute to the IFN response (Supplementary Figure 6). AFF3 and H19 transcript levels were elevated in SSc fibroblast exosomes compared to healthy control fibroblasts. AFF3 is an immunoglobulin class switch factor that has been shown to be a susceptibility factor for rheumatoid arthritis,^19^ and H19 has been shown to regulate ISG expression in macrophages in response to lipopolysaccharide stimulation.^20^ These two factors may contribute to the type 1 IFN response in the keratinocytes, but it is more likely that the exosomes could contain unshielded/uncapped RNA as described in the CAF work.^4^, ^5^ In this regard, the uncapped RNA (RN7SL1) has been shown to be directly linked to Notch/c‐Myc/RNA Pol III function. We have shown Notch signaling is enhanced in SSc,^24^, ^25^ and RNA Pol III has been implicated previously.^26^ This suggests the exosome RNA from SSc fibroblasts may lead to the activation of the type I IFN pathway in the keratinocytes through recognition of uncapped RNA structures. Therefore, future work will focus on proteomic and RNA‐seq analysis of SSc fibroblast exosome cargo to determine if cytokines or the specific RNA transcript RN7SL1 are present in the exosomes. Exosomes are known carriers of miRNA, and a number of miRNA are known to regulate type I IFN responses. Furthermore, a number of differentially expressed long noncoding RNAs in SSc have been implicated in regulating IFN responses.^11^ Therefore, it is possible that a number of miRNA or long noncoding RNA within the exosomes may regulate the responses we have observed in the keratinocytes.

Interestingly, the RNA‐seq data revealed that healthy fibroblast exosomes have an anti‐inflammatory effect on HaCaTs that is lost in SSc fibroblast exosomes, which have a counteractive effect. This sheds a new light on the possible role of healthy fibroblasts exosomes in regulating the immune response to maintain skin homeostasis, whereas this homeostatic regulation is disrupted with SSc fibroblast exosomes. When healthy fibroblast exosomal RNA was isolated and transfected into keratinocytes, the RNA did not impart an anti‐inflammatory effect (Figure 5F–H) as observed in the RNA‐seq analysis (Figure 3) This suggests proteins within the healthy fibroblast exosomes may impart the anti‐inflammatory response, but the RNA from SSc fibroblast exosomes reverses this effect.

The ability of the JAK inhibitor tofacitinib to prevent SSc fibroblast exosome–mediated ISG expression is intriguing. These data suggest exosome‐mediated ISG expression is triggered by cytokine receptor–mediated activation of STAT1. The precise cytokine receptor involved is still unknown (Figure 6). There are a number of receptors that may play a role including the IL‐6 and IFN‐1 receptor. Future work will involve testing the supernatants of exosome stimulated keratinocytes for candidate stimulatory cytokines.

An exciting element of the tofacitinib data is that this drug is approved in the clinic for rheumatoid and psoriatic arthritis. Early clinical trials in SSc have had promising outcomes.^12^ Tofacitinib blocked ISG signatures in both fibroblasts and keratinocytes. Our data follow those produced in the trial and add an extra layer of mechanistic data by suggesting the compound is blocking the effects of the ISG signature driven through the crosstalk between these cells.

Bhandari et al showed that SSc fibroblast exosomes can induce macrophage activation.^9^ This observation is complementary to our data showing the same exosomes can induce a type 1 IFN response in keratinocytes. It remains to be determined what factor(s) within the exosomes lead to the activation of the macrophages and keratinocytes. Therefore, it would be interesting to investigate if the RNA contained within the exosomes can induce the response in macrophages. In addition, it would be interesting to investigate if the supernatant from the exosome stimulated macrophages could induce a response in the keratinocytes and vice versa. This could result in an immune signaling cascade in patient skin with the fibroblast exosomes as the culprit of this cascade. Bhandari et al showed the supernatant from exosome stimulated macrophages could induce myofibroblast activation in naïve fibroblasts. This suggests the exosomes induce a potent response in the macrophages. Unfortunately, from the study we were unable to conclude if the healthy dermal fibroblast exosomes imparted an anti‐inflammatory response in the macrophages (similar to the keratinocytes), as the authors did not show a comparison between unstimulated macrophages and macrophages stimulated with healthy fibroblast exosomes.

In conclusion, we have proposed a mechanism behind the type 1 IFN response found is SSc skin keratinocytes, and our experimental data suggest that tissue fibroblasts may be at least one of the sources of type I IFN activation observed in SSc.

## AUTHOR CONTRIBUTIONS

All authors contributed to at least one of the following manuscript preparation roles: conceptualization AND/OR methodology, software, investigation, formal analysis, data curation, visualization, and validation AND drafting or reviewing/editing the final draft. As corresponding author, Dr Del Galdo confirms that all authors have provided the final approval of the version to be published, and takes responsibility for the affirmations regarding article submission (eg, not under consideration by another journal), the integrity of the data presented, and the statements regarding compliance with institutional review board/Declaration of Helsinki requirements.

## ROLE OF THE STUDY SPONSOR

Boehringer Ingelheim colleagues had a role in the study design or in the collection, analysis, or interpretation of the data, the writing of the manuscript, or the decision to submit the manuscript for publication. Publication of this article was contingent upon approval by Boehringer Ingelheim.

## Supporting information


Disclosure form

**Supplementary Figure 1:** Scleroderma dermal fibroblasts induce type I interferon signalling in keratinocytes.

**Supplementary Figure 2:** SSc fibroblast exosomes impart a pro‐inflammatory effect on keratinocytes compared to Healthy control fibroblast exosomes.

**Supplementary Figure 3:** SSc fibroblast‐derived exosomes induce interferon‐stimulated genes and pSTAT1 in keratinocytes, after 48 hours.

**Supplementary Figure 4:** Scleroderma dermal fibroblasts exosomes induce Type 1 IFN signalling in keratinocytes independent of IRF3/7.

**Supplementary Figure 5:** Inhibition of Tank‐binding Kinase and JAK‐STAT blocks SSc fibroblast exosome mediated Type I IFN signalling in keratinocytes.

**Supplementary Figure 6:** Analysis of mRNA content between healthy and SSc fibroblast exosomes.

Supplementary Table 1:

**Appendix S1:** Materials and Methods.

**Appendix S2:** Supporting Information.
