# Supplementary material for: Systemic Sclerosis Dermal Fibroblast Exosomes Trigger Type 1 Interferon Responses in Keratinocytes via a TBK/JAK/STAT Signaling Axis
Source: Arthritis Rheumatol. 2024 Nov 12;77(3):322–34. doi: 10.1002/art.43029 (PMC11865698; doi:10.1002/art.43029)

Supplementary Figure 1 :Scleroderma dermal fibroblasts induce type I interferon signalling in keratinocytes.

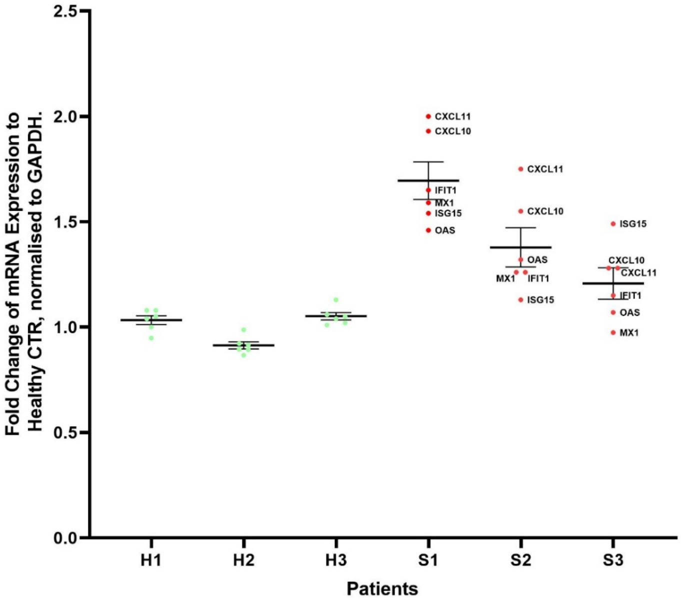

Supplement: Supplementary file 2 — Supplementary Figure 1: Scleroderma dermal fibroblasts induce type I interferon signalling in keratinocytes. [file ART-77-322-s002.pdf]
