# Supplementary material for: Systemic Sclerosis Dermal Fibroblast Exosomes Trigger Type 1 Interferon Responses in Keratinocytes via a TBK/JAK/STAT Signaling Axis
Source: Arthritis Rheumatol. 2024 Nov 12;77(3):322–34. doi: 10.1002/art.43029 (PMC11865698; doi:10.1002/art.43029)
Supplement: Supplementary file 3 — Supplementary Figure 2: SSc fibroblast exosomes impart a pro‐inflammatory effect on keratinocytes compared to Healthy control fibroblast exosomes. [file ART-77-322-s006.pdf]

Supplementary Figure 2: SSc fibroblast exosomes impart a pro-inflammatory effect on keratinocytes compared to Healthy control fibroblast exosomes

Gene	HC vs Ctrl FC	HC vs Ctrl P	SSc vs Ctrl FC	SSc vs Ctrl P	SSc vs HC FC	SSc vs HC P
IL7	-2.7	0.0050	-0.6	0.3898	2.1	0.0232
CXCL6	-1.5	0.0190	-0.2	0.7231	1.3	0.0254
CXCL1	-1.6	0.0035	-0.6	0.1875	1	0.0499
GBP1	-0.7	0.0005	0.0	0.8895	0.8	0.0001
MMP7	-0.5	0.0432	0.1	0.5564	0.6	0.0050
TREM2	-1.3	0.0478	0.0	0.9734	1.3	0.0276
CTSS	-0.9	0.0082	-0.2	0.4660	0.6	0.0276
