# Supplementary material for: Systemic Sclerosis Dermal Fibroblast Exosomes Trigger Type 1 Interferon Responses in Keratinocytes via a TBK/JAK/STAT Signaling Axis
Source: Arthritis Rheumatol. 2024 Nov 12;77(3):322–34. doi: 10.1002/art.43029 (PMC11865698; doi:10.1002/art.43029)
Supplement: Supplementary file 4 — Supplementary Figure 3: SSc fibroblast‐derived exosomes induce interferon‐stimulated genes and pSTAT1 in keratinocytes, after 48 hours. [file ART-77-322-s009.pdf]

Supplementary Figure 3: SSc fibroblast-derived exosomes induce interferon-stimulated genes and pSTAT1 in keratinocytes, after 48 hours.

Gene	Fold Change<br>Log2	p value<br>–log10
ISG15	2.21822844	1.586692523
MX1	1.765299061	1.965958378
OAS1	1.736810579	2.171046056
OAS2	1.722130462	1.684541185
TAP1	1.683017546	2.572721675
BST2	1.572758062	1.394140898
ADAR	1.421541165	1.935286751
IRF9	1.420463895	1.953597167
IFI30	1.369560648	1.48804029
PML	1.362108826	2.033843431
STAT2	1.228020718	1.963525251
IRF2	1.174936835	2.612926933
TICAM1	1.156257236	2.233192006
IFI6	1.146502477	1.338225835
SHB	1.016542773	2.009535582
TRAF3	0.41803364	1.605047733
