# Supplementary material for: Systemic Sclerosis Dermal Fibroblast Exosomes Trigger Type 1 Interferon Responses in Keratinocytes via a TBK/JAK/STAT Signaling Axis
Source: Arthritis Rheumatol. 2024 Nov 12;77(3):322–34. doi: 10.1002/art.43029 (PMC11865698; doi:10.1002/art.43029)

Supplementary Figure 4 :Scleroderma dermal fibroblasts exosomes induce Type 1 IFN signalling in keratinocytes independent of IRF3/7

A

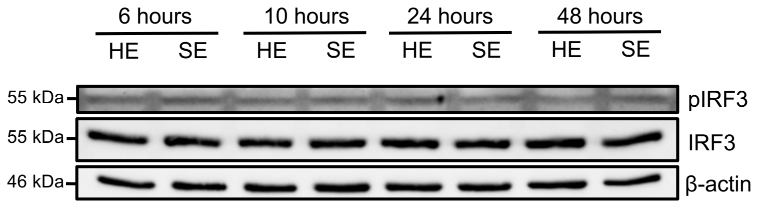

B

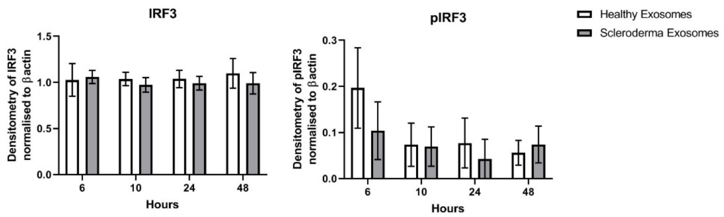

Supplement: Supplementary file 5 — Supplementary Figure 4: Scleroderma dermal fibroblasts exosomes induce Type 1 IFN signalling in keratinocytes independent of IRF3/7. [file ART-77-322-s004.pdf]
