# Supplementary material for: Systemic Sclerosis Dermal Fibroblast Exosomes Trigger Type 1 Interferon Responses in Keratinocytes via a TBK/JAK/STAT Signaling Axis
Source: Arthritis Rheumatol. 2024 Nov 12;77(3):322–34. doi: 10.1002/art.43029 (PMC11865698; doi:10.1002/art.43029)
Supplement: Supplementary file 8 — Supplementary Table 1: [file ART-77-322-s005.docx]

| **GeneID** | **Symbol** | **Description** | **log2 fold change** | **P-value** |
| --- | --- | --- | --- | --- |
| ENSG00000104432 | IL7 | interleukin 7 [Source:HGNC Symbol;Acc:HGNC:6023] | 2.05 | 0.023178295 |
| ENSG00000263818 | RDM1P5 | RDM1 pseudogene 5 [Source:HGNC Symbol;Acc:HGNC:53921] | 1.91 | 0.003695791 |
| ENSG00000159860 | TCAF2P1 | TRPM8 channel associated factor 2 pseudogene 1 [Source:HGNC Symbol;Acc:HGNC:33603] | 1.75 | 0.006250085 |
| ENSG00000112394 | SLC16A10 | solute carrier family 16 member 10 [Source:HGNC Symbol;Acc:HGNC:17027] | 1.75 | 0.065223947 |
| ENSG00000064787 | BCAS1 | brain enriched myelin associated protein 1 [Source:HGNC Symbol;Acc:HGNC:974] | 1.69 | 0.014457407 |
| ENSG00000112309 | B3GAT2 | beta-1,3-glucuronyltransferase 2 [Source:HGNC Symbol;Acc:HGNC:922] | 1.62 | 0.009008074 |
| ENSG00000128849 | CGNL1 | cingulin like 1 [Source:HGNC Symbol;Acc:HGNC:25931] | 1.58 | 0.004705505 |
| ENSG00000138411 | HECW2 | HECT, C2 and WW domain containing E3 ubiquitin protein ligase 2 [Source:HGNC Symbol;Acc:HGNC:29853] | 1.56 | 0.033262326 |
| ENSG00000165807 | PPP1R36 | protein phosphatase 1 regulatory subunit 36 [Source:HGNC Symbol;Acc:HGNC:20097] | 1.52 | 0.003632995 |
| ENSG00000174950 | CD164L2 | CD164 molecule like 2 [Source:HGNC Symbol;Acc:HGNC:32043] | 1.51 | 0.013393943 |
| ENSG00000105409 | ATP1A3 | ATPase Na+/K+ transporting subunit alpha 3 [Source:HGNC Symbol;Acc:HGNC:801] | 1.48 | 0.012145908 |
| ENSG00000260101 | AFTPH-DT | AFTPH divergent transcript [Source:HGNC Symbol;Acc:HGNC:52788] | 1.48 | 0.004199333 |
| ENSG00000162078 | ZG16B | zymogen granule protein 16B [Source:HGNC Symbol;Acc:HGNC:30456] | 1.46 | 0.000997203 |
| ENSG00000228295 | LINC00392 | long intergenic non-protein coding RNA 392 [Source:HGNC Symbol;Acc:HGNC:42720] | 1.44 | 0.026275817 |
| ENSG00000279133 | | TEC | 1.44 | 0.010105173 |
| ENSG00000015413 | DPEP1 | dipeptidase 1 [Source:HGNC Symbol;Acc:HGNC:3002] | 1.43 | 0.02586371 |
| ENSG00000178568 | ERBB4 | erb-b2 receptor tyrosine kinase 4 [Source:HGNC Symbol;Acc:HGNC:3432] | 1.35 | 0.014636453 |
| ENSG00000124875 | CXCL6 | C-X-C motif chemokine ligand 6 [Source:HGNC Symbol;Acc:HGNC:10643] | 1.30 | 0.025387939 |
| ENSG00000095970 | TREM2 | triggering receptor expressed on myeloid cells 2 [Source:HGNC Symbol;Acc:HGNC:17761] | 1.27 | 0.027568807 |
| ENSG00000176040 | TMPRSS7 | transmembrane serine protease 7 [Source:HGNC Symbol;Acc:HGNC:30846] | 1.26 | 0.005688001 |
| ENSG00000184351 | KRTAP19-1 | keratin associated protein 19-1 [Source:HGNC Symbol;Acc:HGNC:18936] | 1.22 | 0.03135344 |
| ENSG00000168952 | STXBP6 | syntaxin binding protein 6 [Source:HGNC Symbol;Acc:HGNC:19666] | 1.20 | 0.018117115 |
| ENSG00000130433 | CACNG6 | calcium voltage-gated channel auxiliary subunit gamma 6 [Source:HGNC Symbol;Acc:HGNC:13625] | 1.18 | 0.002064027 |
| ENSG00000164125 | GASK1B | golgi associated kinase 1B [Source:HGNC Symbol;Acc:HGNC:25312] | 1.18 | 0.092943352 |
| ENSG00000233175 | FMNL1-AS1 | FMNL1 antisense RNA 1 [Source:HGNC Symbol;Acc:HGNC:55717] | 1.18 | 0.067660072 |
| ENSG00000272183 | | novel transcript, antisense to LBX2 | 1.17 | 0.038391834 |
| ENSG00000152611 | CAPSL | calcyphosine like [Source:HGNC Symbol;Acc:HGNC:28375] | 1.15 | 0.007229255 |
| ENSG00000145569 | OTULINL | OTU deubiquitinase with linear linkage specificity like [Source:HGNC Symbol;Acc:HGNC:25629] | 1.12 | 0.052187344 |
| ENSG00000280339 | | novel transcript | 1.12 | 0.017820691 |
| ENSG00000267938 | EIF1P6 | eukaryotic translation initiation factor 1 pseudogene 6 [Source:HGNC Symbol;Acc:HGNC:49619] | 1.12 | 0.016644879 |
| ENSG00000128917 | DLL4 | delta like canonical Notch ligand 4 [Source:HGNC Symbol;Acc:HGNC:2910] | 1.12 | 0.063967698 |
| ENSG00000271404 | MZT1P2 | mitotic spindle organizing protein 1 pseudogene 2 [Source:HGNC Symbol;Acc:HGNC:50606] | 1.10 | 0.077714358 |
| ENSG00000283098 | | novel transcript | 1.10 | 0.067239781 |
| ENSG00000120068 | HOXB8 | homeobox B8 [Source:HGNC Symbol;Acc:HGNC:5119] | 1.10 | 0.04039683 |
| ENSG00000224689 | ZNF812P | zinc finger protein 812, pseudogene [Source:HGNC Symbol;Acc:HGNC:33242] | 1.10 | 0.040629891 |
| ENSG00000165887 | ANKRD2 | ankyrin repeat domain 2 [Source:HGNC Symbol;Acc:HGNC:495] | 1.08 | 0.051405899 |
| ENSG00000226904 | | novel transcript | 1.07 | 0.031593272 |
| ENSG00000280388 | | TEC | 1.07 | 0.134961778 |
| ENSG00000071909 | MYO3B | myosin IIIB [Source:HGNC Symbol;Acc:HGNC:15576] | 1.07 | 0.015571813 |
| ENSG00000261135 | | novel transcript | 1.05 | 0.024062971 |
| ENSG00000182218 | HHIPL1 | HHIP like 1 [Source:HGNC Symbol;Acc:HGNC:19710] | 1.04 | 0.023093468 |
| ENSG00000224596 | ZMIZ1-AS1 | ZMIZ1 antisense RNA 1 [Source:HGNC Symbol;Acc:HGNC:27433] | 1.04 | 0.042384486 |
| ENSG00000177335 | LINC02904 | long intergenic non-protein coding RNA 2904 [Source:HGNC Symbol;Acc:HGNC:26731] | 1.03 | 0.010987831 |
| ENSG00000215068 | ANXA2R-AS1 | ANXA2R antisense RNA 1 [Source:HGNC Symbol;Acc:HGNC:27208] | 1.03 | 0.003381544 |
| ENSG00000221845 | LINC02902 | long intergenic non-protein coding RNA 2902 [Source:HGNC Symbol;Acc:HGNC:34432] | 1.02 | 0.079033526 |
| ENSG00000108984 | MAP2K6 | mitogen-activated protein kinase kinase 6 [Source:HGNC Symbol;Acc:HGNC:6846] | 1.02 | 0.040765382 |
| ENSG00000163739 | CXCL1 | C-X-C motif chemokine ligand 1 [Source:HGNC Symbol;Acc:HGNC:4602] | 1.02 | 0.049908206 |
| ENSG00000272008 | | novel transcript, antisense to ZNF292 | 1.02 | 0.028438086 |
| ENSG00000271828 | | novel transcript, antisense to MIER3 | 1.01 | 0.033710318 |
| ENSG00000175600 | SUGCT | succinyl-CoA:glutarate-CoA transferase [Source:HGNC Symbol;Acc:HGNC:16001] | 1.01 | 0.083286199 |
| ENSG00000069764 | PLA2G10 | phospholipase A2 group X [Source:HGNC Symbol;Acc:HGNC:9029] | 1.00 | 0.051332555 |
| ENSG00000127920 | GNG11 | G protein subunit gamma 11 [Source:HGNC Symbol;Acc:HGNC:4403] | 1.00 | 0.254453441 |
| ENSG00000144229 | THSD7B | thrombospondin type 1 domain containing 7B [Source:HGNC Symbol;Acc:HGNC:29348] | 1.00 | 0.010080226 |
| ENSG00000104055 | TGM5 | transglutaminase 5 [Source:HGNC Symbol;Acc:HGNC:11781] | 1.00 | 0.018856011 |
| ENSG00000279611 | | novel transcript | 0.99 | 0.284653693 |
| ENSG00000272989 | LINC02012 | long intergenic non-protein coding RNA 2012 [Source:HGNC Symbol;Acc:HGNC:52847] | 0.99 | 0.076241418 |
| ENSG00000173567 | ADGRF3 | adhesion G protein-coupled receptor F3 [Source:HGNC Symbol;Acc:HGNC:18989] | 0.99 | 0.021093173 |
| ENSG00000254428 | | novel transcript, antisense to CCDC84 | 0.99 | 0.116592866 |
| ENSG00000183091 | NEB | nebulin [Source:HGNC Symbol;Acc:HGNC:7720] | 0.99 | 0.247606266 |
| ENSG00000123500 | COL10A1 | collagen type X alpha 1 chain [Source:HGNC Symbol;Acc:HGNC:2185] | 0.98 | 0.017990394 |
